# Supplementary material for: Decoding the effects of synonymous variants
Source: Nucleic Acids Res. 2021 Nov 30;49(22):12673–91. doi: 10.1093/nar/gkab1159 (PMC8682775; doi:10.1093/nar/gkab1159)
Supplement: gkab1159_Supplemental_Files [file gkab1159_supplemental_files.zip › supp_tables.pdf]

Supplementary Tables

Supplementary Table S1. ***Curated-effect*** **sSNVs.**

Chr	Position	Ref	Alt	Source	Disease	Effect	Gene
5	112170773	G	T	SilVA (1)	familial adenomatous polyposis	exon loss	APC
X	66943587	C	T		androgen insensitivity syndrome	splice site activation	AR
11	108151895	G	A		ataxia telangiectasia	exon skip	ATM
20	44751769	A	T		X-linked hyper IgM syndrome	exon loss	CD40
7	117243607	G	T		cystic fibrosis	create splice site	CFTR
17	4804090	G	A		congenital myasthenic syndrome	create splice site	CHRNE
2	219674479	G	T		cerebrotendinous xanthomatosis	activate splice site	CYP27A1
X	138630589	G	A		Hemophilia B	unknown	F9
15	48729544	G	A		Tyrosinemia type 1	exon loss	FAH
15	48729544	G	A		Marfan Syndrome	exon loss	FBN1
10	123263444	C	T		Crouzon Syndrome	splice site activation	FGFR2
15	72645409	C	T		Tay Sachs	exon loss	HEXA
11	118958997	C	G		acute intermittent porphyria	exon loss	HMBS
X	133632702	C	T		Lesch Nyhan Syndrome	exon loss	HPRT1
17	45368454	G	A		Glanzmann thrombasthenia	exon loss	ITGB3
10	90982268	C	T		cholesteryl ester storage disease	exon skip	LIPA
17	44087705	T	C		frontotemporal dementia with parkinsonism	increase exon inclusion	MAPT
17	44061058	T	C		familial dementia	increase exon inclusion	MAPT
17	44087768	T	C		progressive supranuclear palsy	increase exon inclusion	MAPT
3	37083822	G	A		Lynch syndrome	exon loss	MLH1
17	29527613	G	A		neurofibromatosis type 1	exon loss	NF1
12	103240673	T	C		Phenylketonuria	increase exon inclusion	PAH

12	103237426	T	A		Phenylketonuria	exon loss	PAH
X	19373511	A	G		Leigh's syndrome	exon loss	PDHA1
1	155263229	C	T		pyruvate kinase deficiency	exon loss	PKLR
11	112101405	G	A		PTPS deficiency	exon loss	PTS
10	43609989	C	T		Hirschsprung disease	aberrant splicing	RET
5	70247773	C	T		spinal muscular atrophy	exon loss	SMN1
5	149772946	A	C		Treacher Collins Syndrome	exon loss	TCOF1
17	7578195	C	T		Cancer susceptibility	intron retention	TP53
X	47065502	C	T		X linked infantile spinal muscular atrophy	expression reduction	UBA1
1	45480678	G	A		familial porphyria cutanea tarda	exon loss	UROD
19	39898667	C	T		Cancer progression	translational efficiency decrease	ZFP36
1	161276637	C	A	(2)	Charcot Marie Tooth disease type1B	aberrant splicing	MPZ
9	36223374	T	C	(3)	GNE myopathy	aberrant splicing	GNE
1	156105820	G	A	(4)	autosomal dominant cardiomyopathy	aberrant splicing	LMNA
1	149898428	G	A	(5)	Nager syndrome	aberrant splicing	SF3B4
5	35867519	T	A	(6)	severe combined immunodeficiency	aberrant splicing	IL7R
11	31823428	G	A	(7)	congenital aniridia	exon shortening	PAX6
13	52511419	A	T	(8)	Wilson disease	exon skip	ATP7B
1	161276535	G	A	(9)	Charcot Marie Tooth disease type1B	splice site activation	MPZ
19	50169131	C	T	(10)	melanoma	miRNA affinity reduction	BCL2L12

Supplementary Table S2. Features used in building machine learning models. Feature ID is the column name in training data, corresponding to a feature name described in methods. The features can be categorized in different groups. Explanation and data type of the features are also presented.

Feature ID	Feature name	Group	Note	Data type
d_fracOpt	ΔfracOpt	codon bias	fraction of optimal codon, difference due to variant	continuous
codon_mutation	codon_mutation	other	codon to codon mutation (e.g. CCC>CCT)	categorical
d_tAI	ΔtAI	codon bias	tRNA adaptation index, difference due to variant	continuous
d_cais	ΔCAI	codon bias	codon adaptation index, difference due to variant	continuous
d_ICDIs	ΔICDI	codon bias	Instrinsic codon deviation index, difference due to variant	continuous
next_codon	next codon	other	next codon to the mutated codon	categorical
PREL	solvent accessibility	protein structure	solvent accessibility predicted by PredictProtein	categorical
d	global structural disimilarity	mRNA stability	global structural disimilarity predicted by RNAsnp	continuous
len	Transcript length	other	Transcript length	continuous
GC	GC content	other	GC content	continuous
dmax	local structural disimilarity	mRNA stability	local structural disimilarity at optimal sequence interval predicted by RNAsnp	continuous
cais	CAI	codon bias	codon adpation index	continuous
local_mRNAstruc	local mRNA structure	mRNA stability	local mRNA structure (upstream/downstream weakly/strongly paired/unpaired) predicted by RNAfold	categorical
d_Bs	ΔCUB	codon bias	codon usage bias, difference due to variant	continuous
freq_change	FreqChange	autocorrelation	change of frequency of original/substituted codon before/after mutation	continuous
tAI	tAI	codon bias	tRNA adaptation index	continuous

d_scuos	ΔSCUO	codon bias	synonymous codon usage order, difference due to variant	continuous
TPI2	CAM	autocorrelation	codon autocorrelation measure	continuous
dist_tfbs	distance to TFBS	distance to regulatory factors	distance to transcript factor binding site	categorical
MD2st	protein local disorderedness	protein structure	binary prediction from PredictProtein: disordered or non-disordered	binary
dist_splice	distance to splice sites	distance to regulatory factors	distance to splice sites	categorical
pos1	relative position	other	relative position of the variant in the transcript	continuous
struc_freq	structural frequency	mRNA stability	frequency of the Minimum Free Energy structure predicted by RNAfold	continuous
centroid_energy	centroid engergy	mRNA stability	transcript centroid engergy predicted by RNAfold	continuous
PHEL	Protein secondary structure	protein structure	Protein secondary structure (helix/sheet/loop) at the variant	categorical
last_codon	previous codon	other	previous codon to the mutated codon	categorical
centroid_distance	centroid distance	mRNA stability	distance of possible structures to centroid structure predicted by RNAfold	continuous
struc_diversity	structural diversity	mRNA stability	transcript structural diversity predicted by RNAfold	continuous
dist_rpb	distance to RBP	distance to regulatory factors	distance to RNA binding protein motifs	categorical
log10MinExp	log10(Min. Expression)	expression profile	log10(Min. Expression)	continuous
strand	genomic coding strand	other	whether the coding sequencing is located at positive or negative strand	binary

dist_esr	distance to ESR	distance to regulatory factors	distance to exonic splicing regulatory motifs	categorical
log10MedianExp	log10(Median Expression)	expression profile	log10(Median Expression)	continuous
log10MaxExp	log10(Max. Expression)	expression profile	log10(Max. Expression)	continuous
mRNAStrucChange	mRNA structural change	mRNA stability	local mRNA structural change, identified by comparing RNAfold predictions before and after mutation	categorical

Supplementary Table S3. ClinVar *benign* and *pathogenic* sSNVs. PubMed ID for *pathogenic* variants are attached.

chr	pos	ref	alt	Clinical significance	PubMed ID
17	41219707	G	A	benign	
17	41223119	T	C	benign	
17	41234470	A	G	benign	
17	41243864	G	A	benign	
17	41244116	C	T	benign	
17	41244734	T	C	benign	
17	41245237	A	G	benign	
17	41245316	A	G	benign	
17	41245439	T	C	benign	
17	41245466	G	A	benign	
17	41245577	T	C	benign	
17	41246156	G	A	benign	
17	41246567	T	C	benign	
17	41246590	T	G	benign	
17	41246741	C	T	benign	
17	41246753	A	G	benign	
17	41249263	G	A	benign	
17	41258484	A	G	benign	
17	41267763	C	T	benign	
17	41276093	G	A	benign	
17	42452054	C	T	benign	
17	42455126	C	T	benign	
17	42457087	C	T	benign	
17	45360896	T	C	benign	
17	45376885	C	T	benign	
17	78078709	T	C	benign	

17	78081515	G	A	benign	
17	78082504	G	A	benign	
17	78092063	G	A	benign	
19	4090605	G	A	benign	
19	4094469	C	T	benign	
19	4095412	G	A	benign	
19	4099272	G	A	benign	
19	4099293	C	T	benign	
19	4099295	G	A	benign	
19	4101032	C	T	benign	
19	4101062	G	T	benign	
19	4101119	G	A	benign	
19	4101261	C	T	benign	
19	4102377	G	A	benign	
19	4102404	G	A	benign	
19	4102449	G	A	benign	
19	4110537	G	A	benign	
19	4110552	C	G	benign	
19	4117429	G	T	benign	
19	4117495	G	A	benign	
19	4117528	G	A	benign	
19	4117579	G	A	benign	
21	36164486	G	C	benign	
21	36164606	G	A	benign	
21	36164789	C	G	benign	
17	41199683	C	T	pathogenic	
17	41242961	C	T	pathogenic	
1	94564350	C	A	pathogenic	28118664
1	216498841	G	T	pathogenic	20513143

11	2604775	G	A	pathogenic	29857160
13	32954050	G	A	pathogenic	25382762
14	58949430	G	A	pathogenic	26096313
2	47708010	G	A	pathogenic	23523604
3	37042536	C	T	pathogenic	15235038
3	37059088	C	T	pathogenic	26761715
3	37061954	G	A	pathogenic	25525159
3	37083822	G	A	pathogenic	25525159
3	37089174	G	A	pathogenic	22081473
7	117246807	G	A	pathogenic	25066652
7	117254767	G	A	pathogenic	9067754
X	148568514	G	A	pathogenic	27146977
X	153594930	C	T	pathogenic	29024177

References

1. Buske, O.J., Manickaraj, A., Mital, S., Ray, P.N. and Brudno, M. (2013) Identification of deleterious synonymous variants in human genomes. *Bioinformatics*, **29**, 1843-1850.
2. Corrado, L., Magri, S., Bagarotti, A., Carecchio, M., Piscosquito, G., Pareyson, D., Varrasi, C., Vecchio, D., Zonta, A. and Cantello, R. (2016) A novel synonymous mutation in the MPZ gene causing an aberrant splicing pattern and Charcot-Marie-Tooth disease type 1b. *Neuromuscular Disorders*, **26**, 516-520.
3. Zhu, W., Eto, M., Mitsuhashi, S., Takata, K., Beck, G., Sumi-Akamaru, H., Mochizuki, H., Sakoda, S., Takahashi, M.P. and Nishino, I. (2018) GNE myopathy caused by a synonymous mutation leading to aberrant mRNA splicing. *Neuromuscular Disorders*, **28**, 154-157.
4. Ito, K., Patel, P.N., Gorham, J.M., McDonough, B., DePalma, S.R., Adler, E.E., Lam, L., MacRae, C.A., Mohiuddin, S.M. and Fatkin, D. (2017) Identification of pathogenic gene mutations in LMNA and MYBPC3 that alter RNA splicing. *Proceedings of the National Academy of Sciences*, **114**, 7689-7694.
5. Cassina, M., Cerqua, C., Rossi, S., Salviati, L., Martini, A., Clementi, M. and Trevisson, E. (2017) A synonymous splicing mutation in the SF3B4 gene segregates in a family with highly variable Nager syndrome. *European journal of human genetics*, **25**, 371-375.
6. Gallego-Bustos, F., Gotea, V., Ramos-Amador, J.T., Rodríguez-Pena, R., Gil-Herrera, J., Sastre, A., Delmiro, A., Rai, G., Elnitski, L. and González-Granado, L.I. (2016) A case of IL-7R deficiency caused by a novel synonymous mutation and implications for mutation screening in SCID diagnosis. *Frontiers in immunology*, **7**, 443.
7. Filatova, A.Y., Vasilyeva, T.A., Marakhonov, A.V., Voskresenskaya, A.A., Zinchenko, R.A. and Skoblov, M.Y. (2019) Functional reassessment of PAX6 single nucleotide variants by in vitro splicing assay. *European Journal of Human Genetics*, **27**, 488-493.
8. Wang, C., Zhou, W., Huang, Y., Yin, H., Jin, Y., Jia, Z., Zhang, A., Liu, Z. and Zheng, B. (2018) Presumed missense and synonymous mutations in ATP 7B gene cause exon skipping in Wilson disease. *Liver International*, **38**, 1504-1513.
9. Taioli, F., Cabrini, I., Cavallaro, T., Simonati, A., Testi, S. and Fabrizi, G.M. (2011) Déjerine-Sottas syndrome with a silent nucleotide change of myelin protein zero gene. *Journal of the Peripheral Nervous System*, **16**, 59-64.
10. Gartner, J.J., Parker, S.C., Prickett, T.D., Dutton-Regester, K., Stitzel, M.L., Lin, J.C., Davis, S., Simhadri, V.L., Jha, S. and Katagiri, N. (2013) Whole-genome sequencing identifies a recurrent functional synonymous mutation in melanoma. *Proceedings of the National Academy of Sciences*, **110**, 13481-13486.
